# Prevalence and associated risk factors of chronic malnutrition amongst children under five in Eswatini

**DOI:** 10.4102/phcfm.v14i1.3301

**Published:** 2022-08-26

**Authors:** Glorious H. Dlamini, Boikhutso Tlou

**Affiliations:** 1 School of Nursing and Public Health, College of Health Sciences, University of KwaZulu-Natal, Durban, South Africa

**Keywords:** prevalence, malnutrition, children, risk factors, stunting, associated, nutrition

## Abstract

**Background:**

About 20 million children under fi-ve in Southern Africa have chronic malnutrition. This study determines the prevalence of chronic malnutrition and associated risk factors amongst children under five.

**Aim:**

To determine the proportion of children with chronic malnutrition and investigate associated risk factors of chronic malnutrition.

**Setting:**

The study was conducted in communities in the four regions of Eswatini.

**Methods:**

This study is a retrospective cross-sectional study that used data from a Multiple Indicator Cluster Survey (MICS) conducted in 2014. The study involved 3261 children who are under 5 years of age. Data on nutritional status and household characteristics were used. Logistic regression was used to identify factors associated with chronic malnutrition in the univariable and multivariable models, respectively.

**Results:**

Results showed that 18.1% (confidence interval [CI]: 18.5–21.5) of children under five have chronic malnutrition. The highest prevalence was 20.8% (CI: 17.4–24.7) in the Shiselweni region, followed by the Manzini region with 17.6% (CI: 14.5–21.1) and the Lubombo region with 17.2% (CI: 13.9–21.2). The lowest prevalence of chronic malnutrition at 16.7% (CI: 13.6–20.3) was the Hhohho region. The results indicated that low birth weight (odds ratio [OR] = 4.63, CI: 1.12–19.2), mothers’ lack of education (OR = 1.50, CI: 1.04–2.17) and children aged 12–24 months (OR = 1.28, CI: 0.88–1.87) were significantly associated with chronic malnutrition.

**Conclusion:**

The findings showed that malnutrition is an important public health problem in children under five and needs a multisectoral response. Low birth weight, mothers’ education and the child’s age are risk factors associated with chronic malnutrition.

**Contribution:**

The results inform evidence-based programming for the prevention of chronic malnutrition in children thus assist the country to meet sustainable development goals.

## Introduction

The survival of children has improved significantly in the past 50 years.^1^ There has been a decrease in the global under-five death rate by almost 60%, from 93 deaths per 1000 in 1990 to 38 in 2019.^2^ Even with that improvement, about 5.2 million children died before their fifth birthday in 2019 alone.^2^ Sub-Saharan Africa remains one of the regions with the highest mortality rate in children under-five across the world, with one in 13 children dying before their fifth birthday.^2^ Thus, only slight progress has been noted in the poorest regions, especially in sub-Saharan Africa and South Asia.^1^ Contrary to global trends, in sub-Saharan Africa, chronic malnutrition is still persistent in developing countries with major implications on health, particularly amongst poor and vulnerable population groups.^3^

Chronic malnutrition is defined as a form of growth failure which causes both cognitive and physical delays in growth and development.^4^ Stunting, also referred as linear growth failure, is the inability to attain potential height for a particular age, and it is a common measurement used to identify chronic malnutrition.^4^ Stunting is only one manifestation of chronic malnutrition.^5^

Stunting in children has reduced globally from 165.8 million in 2012 to 149 million in 2018, which is a significant 10% decrease.^6^ For the last 25 years, the number of stunted children in the Eastern and Southern African regions has increased from 23.6 million to 26.8 million.^1^ The most affected countries with a high prevalence of chronic malnutrition in young children include Burundi, Uganda and Malawi, with 57.5%, 52.8% and 47.0%, respectively.^7^

According to various cross-sectional studies conducted in Eswatini, chronic malnutrition in children under five has remained above the 15% target set by the World Health Assembly (WHA), over the past 10 years.^8^ It is worth noting that 38% of the most affected children are the orphaned and vulnerable.^9^ The studies also revealed that a high prevalence of chronic malnutrition is experienced in two of the four regions in the country, namely the Shiselweni and Lubombo regions.^9^

The mortality rates in infants and children under five (per 1000 live births) are 85 and 102 deaths, respectively.^2^ The Lubombo region has the highest mortality of 115/1000 live births when compared with other regions, namely Manzini with 112/1000, Shiselweni with 100/1000 and Hhohho with 96/1000.^2^ This translates to a number of children born in Eswatini dying before reaching their fifth birthday and malnutrition is said to be one of the causes of this high mortality.^10^

The Cost of Hunger Report indicated that every year, Eswatini has lost an estimated $92 million as a result of child under-nutrition, which is equivalent to 3.1% of the gross domestic product (GDP), a loss that will recur annually if chronic malnutrition prevalence is not addressed and decreased.^11^ There have been a number of studies conducted focusing only on the prevalence of several health conditions in the country, including malnutrition. However, there are still gaps in understanding the causes of these conditions, including chronic malnutrition, in the context of Eswatini. Therefore, the knowledge to be gathered in this research will bring in-depth understanding on malnutrition’s risk factors for children under five, and it is critical for an evidence-based nutrition response, which will include proper prioritisation and coordination of nutrition interventions.

Furthermore, in recognition of the large disparities in the prevalence of chronic malnutrition, the WHA 2012 has set targets for infant, young child and maternal nutrition, the first of which is to reduce by 40% the number of children under five years of age who have chronic malnutrition by 2025.^12^ Thus, this study will first assist in determining the current prevalence of chronic malnutrition, which will then enable the country to have a baseline. In addition to the set targets by the WHA, Eswatini is also a signatory of the United Nations’ (UN) 2015 sustainable development goals (SDGs). Therefore, understanding the risk factors associated with malnutrition will enable the country to implement evidence-based programmes which will contribute to the second goal of ending all forms of hunger and the third goal of ensuring good health and well-being.^13^

Worth noting is that the country has only been tracking the prevalence of chronic malnutrition, without further analysis of the risks associated with it. Therefore, this is the first study to identify factors associated with chronic malnutrition.

This will contribute to the development of policies and strategies to curb the increasing prevalence of stunting. The results will also influence the design of appropriate programmes that will assist in meeting the international goals set by the WHA and the UN.

## Methodology

### Study design

This study is a retrospective cross-sectional study that used data from a Multiple Indicator Cluster Survey (MICS) conducted in 2014 in Eswatini.

### Study setting

The study was conducted in Eswatini, previously referred to as Swaziland. Eswatini is a landlocked country located in Southern Africa, bordered by South Africa and Mozambique. The country is highly dependent on subsistence farming and is part of the Common Market for Eastern and Southern Africa (COMESA), the African Union and the Southern African Development Community (SADC), to name a few.

Eswatini has a small economy and is classified as a developing country with a GDP per capita of $4145.97, therefore being categorised as a lower-middle income country. Most of the country’s employment is provided by its manufacturing and agricultural sectors and major overseas trading partners, the European Union and the United States of America. However, the youth unemployment rate is currently 47.37%.

### Study population and sampling

Eswatini has a population of about 1 093 238 people and a growth rate of 1.2%, with 29.6% of the people living in urban areas. It is worth noting that the country comprises about 146 348 children below the age of five, and the population of women of childbearing age stands at 7% of the total population. A multistage technique was applied in the sampling of 3261 children under five from 347 enumeration areas (EAs) in the four regions of the country.

The country was stratified into four regions (Hhohho, Shiselweni, Lubombo and Manzini) and two settlement types (urban and rural) in each region. Using the Population and Housing Census 2007 as the sampling frame, a total of 347 EAs were randomly selected across the domains (four regions). The number of EAs selected was decided in order to achieve a given level of precision for a key indicator (in this case, chronic malnutrition). Within each domain, EAs were selected using a systematic selection technique with probability proportional to size (PPS). For each region, the clusters were distributed to the rural and urban domains proportionally to that of the rural and urban population size of that region.

The households were listed alphabetically from A to N by the Central Statistical Office, where 15 households were selected in each EA using a random systematic selection technique. The total sample size for the MICS was 5211 households, where 3261 children under five were found in the households.

### Calculation of sample weights

The MICS sample is not self-weighting. Essentially, by allocating equal numbers of households to each of the regions, different sampling fractions were used in each region, as the population sizes of the regions are different. For this reason, sample weights were calculated, and these were used in the subsequent analyses of the survey data. One of the important components in the calculation of sample weights took into account the level of nonresponse for the household and individual interviews.

### Tools and data collection

The Central Statistics Office under the Ministry of Economic Planning and Development in Eswatini was responsible for coordinating data collection for the MICS in 2014. Trained data collectors were engaged to conduct the door-to-door visit during the collection of the data. The study participants signed consent forms prior to the collection of data. Then, a questionnaire comprising three parts was used for the data collection. The data collected included demographic characteristics, anthropometry screening and assessment of socio-economic and health status of the children. The questionnaire was administered to mothers or caregivers of the children who were under five, living in the household. Households were revisited if there were children living in the household but absent for a maximum of 24 h on the day of data collection. For this research, data were requested from the Ministry of Economic Planning and Development in the department of the Central Statistics Office for further analysis.

### Data analysis

Data were analysed using Statistical Package for the Social Sciences (SPSS) version 27. Univariate analyses were used to summarise demographic characteristics of the study population. Chronic malnutrition was defined according to the World Health Organization (WHO) standards as all children with height-for-age of less than a -2 standard deviation.^12^ Simple logistic regression (unadjusted odds ratio [OR]) was used to determine the individual risk factors related to chronic malnutrition. All statistically significant variables in the univariate analysis were put into the multivariate model for further analysis. Multiple logistic regression was used to identify the factors associated with chronic malnutrition. A *p*-value of less than 0.05 was deemed statistically significant.

### Response variables

In this study, measurements of height in children, taking sex and age into consideration, were transformed into *Z*-scores. Children with a standard deviation below -2 of the WHO median reference for weight-for-height were considered malnourished. Chronic malnutrition was measured as a binary response variable, defined as follows: 1 = normal children and 0 = children with chronic malnutrition.

### Independent variables

Multiple studies and literature reviews were used to select the variables associated with malnutrition. The selection of the variables was also based on their availability in the data sets for the MICS 2014 survey. The United Nations Children’s Fund (UNICEF) and WHO conceptual framework was also used to select potential variables associated with chronic malnutrition. The variables were: sex (1) sex and age of the child, (2) area of residence, (3) mother’s education and age, religion, (4) birth weight, (5) education of head of the household, (6) diarrhea and the main source of drinking water.

### Ethical considerations

Ethical clearance to conduct the study was obtained from the Bio-Medical Research Ethics Committee of the University of KwaZulu-Natal (Clearance No. 00007087). Approval was also granted from Eswatini National Health Research Review Board.

## Results

### Description of the study population

Table 1 presents the demographic characteristics of children under five in four regions of Eswatini, as well as their individual and household characteristics. About 80% of children below 5 years reside in the rural areas in Eswatini, whilst about 20% reside in the urban areas. About 7% of mothers reached tertiary education, and more than 50% (55.4%) have a secondary or high school education. The results also indicated that about 54% of the households use piped water, whilst others use surface water (18.9%), protected wells and boreholes (13.3%) or unprotected (12.9%) water.

**TABLE 1:** Demographic characteristics of children under five in the four regions in Eswatini.

Variable	Categories of explanatory variables	Children under five	
		*n*	%
Child’s sex	Male	1637	50.2
	Female	1637	50.2
Birth weight	Low birth weight	201	16.5
	Normal birth weight	1016	83.5
Age of the child (months)	0–11	659	20.5
	12–23	679	21.2
	24–35	699	21.8
	36–59	1171	35.9
Diarrhoea in the past two weeks	Yes	545	17.0
	No	2654	83.0
Mother’s education	None	255	7.8
	Primary	969	29.8
	Secondary or high school	1801	55.4
	Tertiary	225	6.9
Age of the mother (years)	< 20	664	21.2
	20–34	1751	55.9
	35+	715	22.8
Education of household head	None	699	21.6
	Primary	1169	36.1
	Secondary or high school	1106	34.2
	Tertiary	264	8.2
Area of residence	Rural	2608	80.0
	Urban	653	20.0
Religion	Non-Christian	48	1.5
	Christian	3080	98.5
Main source of drinking water	Surface or rainwater	206	18.9
	Protected well or borehole	146	13.4
	Unprotected well or spring	141	12.9
	Piped water	598	54.8

### The prevalence of chronic malnutrition in children under five

The results indicate that the prevalence of chronic malnutrition was about 18.1% (confidence interval [CI]: 18.5–21.5) at a national level, and the highest prevalence was about 20.8% (CI: 17.4–24.7) in the Shiselweni region, followed by the Manzini and Lubombo regions with about 17.6% (CI: 14.5–21.1) and 17.2% (CI: 13.9–21.2), respectively. The region with the lowest prevalence of chronic malnutrition at 16.7% (CI: 13.6–20.3) was the Hhohho region.

### Risk factors for children under five with chronic malnutrition

Logistic regression was used to assess the factors associated with chronic malnutrition in children under five. A simple logistics regression was performed where only six factors were statistically significant: (1) mother’s education, (2) area of residence, (3) birth weight, (4) age of the child, (5) mother’s education and (6) the education of the household head. Then, a multivariable analysis was performed for all the factors that were statistically significant in the univariable analysis. Birth weight, mother’s education and the child’s age were statistically significant in the multivariate analysis. The results showed that children born to mothers without any formal education were 4.63 times more likely to have chronic malnutrition, compared with those born to mothers with tertiary education (OR = 4.63, CI: 1.12–19.2). The results also indicated that children born to mothers with primary education were 3.83 (OR = 3.83, CI: 0.99–14.74) times more likely to have chronic malnutrition, and those born to mothers with secondary education were 3.32 (OR = 3.32, CI: 0.89–12.41) times more likely to have chronic malnutrition than children born to mothers with a tertiary education. Furthermore, children born with a low birth weight were 1.5 times more likely to have chronic malnutrition than children born with a normal birth weight (OR = 1.50, CI: 1.04–2.17). Furthermore, children within the age group of 0–11 months were approximately 50% less likely to have malnutrition compared to those above 11 months (OR = 0.49, CI: 0.31–0.75), and those aged between 11 and 23 months were 1.3 times more likely to have malnutrition when compared to those aged between 36 and 59 months. However, children aged 12–23 months were 1.28 times (OR = 1.28, CI: 0.88–1.87) more likely to have chronic malnutrition, and children between 24 and 35 months of age were 1.15 times (OR = 1.15, CI: 0.68–1.96) more likely to have chronic malnutrition when compared to children aged 36–59 months.

It is worth noting that the results also indicated that children born to mothers less than the age of 20 years were 1.15 times more likely to have chronic malnutrition when compared to those born to mothers above the age of 35 years (OR = 1.15, CI: 0.65–2.04). However, children born to mothers aged 20–34 years were less likely to have malnutrition when compared to those born to mothers above the age of 35 years (OR = 0.98, CI: 0.63–1.52), even though the results were not statistically significant. Table 2 summarises the univariate and multivariate logistic regression results.

**TABLE 2:** Risk factors associated with chronic malnutrition.

Explanatory variable	Categories of explanatory variable	Univariate analysis			Multivariate analysis		
		Odds ratio	95% confidence interval	*p*	Odds ratio	95% confidence interval	*p*
Birth weight	Low birth weight	1.65	1.16–2.36	0.005	1.50	1.04–2.17	0.030
	Normal weight	1.00	-	-	1.00	-	-
Age of the child (months)	0–11	0.44	0.33–0.60	< 0.0001	0.49	0.31–0.75	0.001
	12–23	1.38	1.10–1.73	0.005	1.28	0.88–1.87	0.204
	24–35	1.19	0.94–1.50	0.145	1.15	0.68–1.96	0.602
	36–59	1.00	-	-	1.00	-	-
Child’s sex	Male	1.15	0.97–1.38	0.123	-	-	-
	Female	1.00	-	-	-	-	-
Diarrhoea in the past 2 weeks	Yes	0.98	0.77–1.24	0.872	-	-	-
	No	1.00	-	-	-	-	-
Age of the mother (years)	< 20	1.26	0.96–1.65	0.009	1.15	0.65–2.04	0.625
	20–34	1.13	0.89–1.42	0.290	0.98	0.63–1.52	0.917
	35+	1.00	-	-	1.00	-	-
Mother’s education	None	6.14	3.22–11.72	< 0.0001	4.63	1.12–19.21	0.035
	Primary	5.70	3.13–10.39	< 0.0001	3.83	0.99–14.74	0.051
	Secondary or higher	3.40	1.87–6.16	< 0.0001	3.323	0.89–12.41	0.074
	Tertiary	1.00	-	-	-	-	-
Education of household head	None	5.84	3.18–10.71	< 0.0001	1.53	0.58–4.02	0.389
	Primary	6.32	3.49–11.65	< 0.0001	1.80	0.71–4.61	0.217
	Secondary or high	3.59	1.96–6.57	< 0.0001	1.29	0.51–3.26	0.585
	Tertiary	1.00	-	-	1.00	-	-
Religion	Non-Christian	0.89	0.41–1.91	0.756	-	-	-
	Christian	1.00	-	-	-	-	-
Main source of drinking water	Surface or rainwater	1.26	0.85–1.87	0.25	-	-	-
	Protected well or borehole	0.99	0.62–1.60	0.99	-	-	-
	Unprotected well or spring	0.67	0.39–1.15	0.15	-	-	-
	Piped water	1.00	-	-	-	-	-
Area of residence	Rural	1.67	1.34–2.15	< 0.0001	0.96	0.65–1.43	0.867
	Urban	1.00	-	-	-	-	-

## Discussion

This study shows that the highest prevalence of chronic malnutrition is in the Shiselweni region with 20.8%, and the Hhohho region has the lowest prevalence rate at 16.7%. This is relatively similar to the MICS 2010 and 2014 studies that also showed the Shiselweni and Hhohho regions with the highest and lowest prevalence, respectively.^9,14^ This confirms the results of an assessment on the Food Security Situation in Swaziland conducted by the Food and Agricultural Organization (FAO), which revealed that the Shiselweni region has the largest prevalence of households with high food insecurity (about 40%), whilst the prevalence is lower in the Hhohho region, estimated at 25%.^15^ The results also reflected an 18.1% prevalence of malnutrition when compared to previous cross-sectional studies, and this is in line with several other studies in the country.^9,14^ The study further investigated factors associated with chronic malnutrition, and the results showed that mother’s education, low birth weight and the child’s age were significant risk factors associated with chronic malnutrition in children under five. Other factors investigated in the study that were not significantly associated with chronic malnutrition are the age of the mother, the area of residence and the level of education of the head of household.

Regarding birth weight, the results indicated that children with a low birth weight were 1.50 times more likely to have chronic malnutrition compared to children born with a normal weight. The association between birth weight and chronic malnutrition is similar to research studies conducted in Bangladesh, Ethiopia and Madagascar, which indicated that children born with a low birth weight were more likely to have chronic malnutrition compared to those born with a normal weight.^16,17,18,19^ It is estimated that intrauterine growth restriction because of maternal under-nutrition accounts for about 20% of the global burden of child malnutrition.^20^ This is probably because of the fact that low birth weight is an indication of inadequate maternal nutrient intake, which has an impact on the growth rate in children. Previous studies have also indicated that children born with a low birth weight are at a higher risk of delayed growth and neurological aberrations and development in their early years.^21^ Furthermore, the association between low birth weight and malnutrition has been found to be aggravated by increased infection in low birth weight children.^22,23^ The researchers suggested that foetal growth restriction compromised antibody generation to polysaccharide antigens, indicating that components of the immune system could be programmed permanently by early life events.

In addition to this, a child’s age was also found to be associated with chronic malnutrition. Children aged between 0 and 11 months are less likely to have chronic malnutrition than those aged 36–59 months, as the results indicated an OR of 0.49 for children aged 0–11 months. This could be because of the fact that children aged 0–11 months are breastfed and those older rely on household food intake for growth and development. Therefore, household food insecurity, unbalanced diets and inadequate meal frequency put children at risk of malnutrition. These results are in line with what has already been found by other research studies conducted in sub-Saharan African countries, which also indicate that the inadequate diets of young children were consistently associated with malnutrition, which is noted in children above 24 months.^16,24^

The results also indicate that children born to uneducated mothers are more likely to have chronic malnutrition when compared to those born to educated mothers. Educated mothers have the ability to make informed choices about appropriate maternal and child nutrition services, including access to health services, particularly antenatal care (ANC) and child health services.^20,25^ This is in line with research conducted in Nairobi, which also found mother’s education to be an important predictor of child malnutrition.^26^ However, other research studies found that the effect of the education of the mother is minimal when compared to factors at maternal, child and household levels.^27,28^

The findings of this research are important for policymakers working on the development of comprehensive nutrition policies in order for the country to meet SDG 2030 targets. Thus, nutrition is important throughout the life cycle and should not be neglected, as it forms part of the growth and development package for children under five. Pregnant mothers should be advised to adhere to good healthy eating practices, which involve sufficient dietary intake through diet diversity in order to meet their nutritional needs and that of the growing foetus, to prevent malnutrition and other related complications in children.^19^

## Conclusion

Despite the substantial progress in reducing malnutrition in children and under-nutrition over the past years, a large number of children under the age of 5 years in Eswatini still fail to achieve their growth potential, as reflected by the high prevalence of malnutrition. This finding showed that malnutrition is an important public health problem in children under five and needs a multisectoral response. The findings also indicate that a low birth weight, mothers’ education and the child’s age are significant risk factors associated with chronic malnutrition. This implies that women’s empowerment should be prioritised not only in the health sector but also across all sectors, including the education sector, as this research indicates children born to less educated mothers are more likely to have chronic malnutrition. For improved nutrition outcomes, empowerment enables mothers to make informed decisions regarding the uptake of maternal and child nutritional services that positively contribute to the growth and development of their children. Promotion and availability of ANC services should be prioritised as a means to improve long-term nutritional and survival status of children. There is a need for the development of policies and strategies for infant and young child feeding to promote dietary diversity in children under five.

### Limitations

The lack of other important variables reported in other studies becomes a limitation for elucidating some important factors. Therefore, the researchers relied on the analysis of only existing variables in the data set. In particular, the absence of socio-economic variables, such as employment status and wealth index, may have decreased the impact of the results. Another limitation is the lack of a control group in the study; therefore, the temporal link between the outcome and the exposure is missing in the findings.
